# Differences in stress response between two altitudes assessed by salivary cortisol levels within circadian rhythms in long-distance runners

**DOI:** 10.1038/s41598-022-13965-w

**Published:** 2022-06-13

**Authors:** Katsuhiko Tsunekawa, Kazumi Ushiki, Larasati Martha, Asuka Nakazawa, Rika Hasegawa, Risa Shimizu, Nozomi Shimoda, Akihiro Yoshida, Kiyomi Nakajima, Takao Kimura, Masami Murakami

**Affiliations:** Department of Clinical Laboratory Medicine, Gunma University Graduate School of Medicine, 3-39-22 Showa-machi Maebashi, Gunma, 371-8511 Japan

**Keywords:** Biomarkers, Endocrinology

## Abstract

There are conflicting reports regarding the efficacy of cortisol as a stress marker in altitude training due to the influence of the circadian rhythm. This study aimed to verify whether the automated measurement of salivary cortisol concentration via sequential sampling could detect the differences in exercise stress between two altitudes. We enrolled 12 elite female long-distance runners living near sea level. For the first higher-altitude camp, the runners lived at 1800 m and trained at 1700 m for 7 days. For the second lower-altitude camp, they lived at 1550 m and trained at 1300 m for 7 days. Their saliva was sequentially collected on the last 2 days during each camp which involved different intensity exercises in the morning and afternoon. The salivary cortisol concentrations were measured using electrochemiluminescence immunoassay. Before dinner, the basal salivary cortisol concentrations were significantly higher in the higher-altitude camp. The rate of change in the salivary cortisol concentration during the morning exercise was significantly higher in the higher-altitude camp than in lower-altitude camp (*p* = 0.028) despite the same exercise programs and intensities. Salivary cortisol level measurements during the athletes' circadian rhythms could detect the differences in acclimatization and exercise stress between two altitudes.

## Introduction

Elite athletes in various sports often train at high altitudes to improve their performance when they return to lower altitudes. At high altitudes with low atmospheric pressure and low oxygen concentrations, the amount of red blood cells and their oxygen-carrying capacity are enhanced by an increase in circulating erythropoietin concentration due to hypoxia-inducible factors^1^. However, a hypoxia during high-altitude training causes excessive stress and decreases an athletes' performance, resulting in poor acclimatization^2^. Therefore, monitoring of physical and psychological stress during exercise training at high-altitude camps may help assess maladaptation.

Cortisol is an important biomarker that is secreted into the circulating plasma from the adrenal cortex via the hypothalamus–pituitary axis as an acute response to stress including exercise^3^. Serum cortisol concentrations are increased by moderate- to high-intensity exercise, but not by low-intensity exercise at less than 40% of athletes' maximal oxygen consumptions (VO_2 max_)^4,5^. However, there have been conflicting reports regarding the efficacy of cortisol as a stress marker during high-altitude training^6–11^, which may be due to the influence of the circadian rhythm. Changes in the serum cortisol concentrations resulting from exercise are greater in the evening than in the morning because of cortisol circadian rhythm^12^. Thus, to accurately evaluate exercise-induced stress using cortisol, it should be measured throughout day. To achieve this, continuous sampling and more efficient cortisol measurement are needed. Serum cortisol concentrations are measured as total hormone conjugated to corticosteroid-binding globulin, whereas salivary cortisol concentrations are measured as free hormones independent of salivary flow rates^13^. Saliva is also advantageous to its ease of collection in the absence of medical professional staff and without the stress of venipuncture^14^. Conventionally, the salivary cortisol concentration has been manually measured using an enzyme-linked immunosorbent assay (ELISA), which this makes it difficult to measure a large number of samples compared with automated methods. Recently, the automated electrochemiluminescence immunoassay (ECLIA) used for serum cortisol concentration measurement was applied to saliva, and the salivary cortisol concentrations measured by ECLIA showed a significantly positive correlation with those measured by liquid chromatography-tandem mass spectrometry^15^ and with conventional ELISA^16^. Moreover, we reported that sequential saliva collection and automated ECLIA-based salivary cortisol measurements could detect the exercise-induced stress within the circadian rhythm in female long-distance runners^16^. In this study, the differences in the rate of change in salivary cortisol concentrations resulting from various exercise intensities could be compared at the same time on different days, even in the early morning.

Training at altitudes of 500–2000 m, defined as low altitude, causes less stress on athletes than training at moderate (2000–3000 m) and high (3000–5500 m) altitudes^17^. In contrast, VO_2 max_ of endurance-trained athletes decreased significantly beginning at 300 m above sea level and continued to decrease linearly by approximately 7% for every 1000 m ascended^18,19^. While these reports suggest that there may be differences in athletes' stress responses at different altitudes, even at low altitudes, few biomarkers have ever detected this difference. We hypothesized that the automated measurement of salivary cortisol concentrations throughout the athletes' circadian rhythms via sequential saliva sampling would enable the assessment of the differences in stress induced by training camps at different altitudes, including those at low altitudes. If proven, this method can help in the prevention of excessive stress, and foster the development of exercise programs in several altitude environments for athletes. The present study expands on our previous study by verifying whether cortisol concentration measurement via continuous saliva collection during the circadian rhythms could adequately detect the differences in acclimatization and stress responses resulting from exercise at different altitudes in female long-distance runners.

## Methods

### Participants

This study was conducted in accordance to the Declaration of Helsinki, and the protocol was approved by the ethics committee of the Gunma University Graduate School of Medicine (Approval number HS2018-140). All the study participants provided written informed consent before being included in the study.

**Figure 1:**
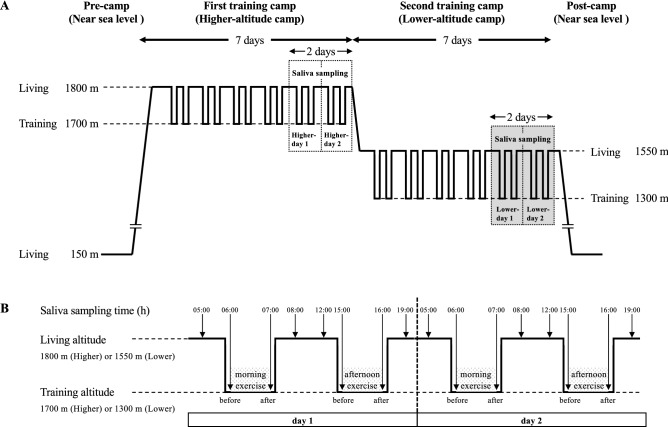
The study design of the first and second training camps of 12 female long-distance runners. The altitudes at which the runners lived and trained during the two training camps and pre- and post-camp (**A**). The schema of altitudes and saliva sampling time in runners on the last 2 days during each training camps (**B**). The downwards arrows denote the saliva sampling from the runners, and the tips of the arrows denote the altitudes at the time of sampling at both camps. Higher-day 1, day 1 at the higher-altitude camp; Higher-day 2, day 2 at the higher-altitude camp; Lower-day 1, day 1 at the lower-altitude camp; Lower-day 2, day 2 at the lower-altitude camp.

We enrolled 12 Japanese elite female long-distance runners. All of them lived in the same dormitory before the first training camp and stayed in the same hotels during camps. Their living conditions, such as wakeup time, meal time, bedtime, and meal content, were standardized before and during the training camps^16^. Figure 1A presents the schedules of the pre-camp and the two training camps. This altitude training was a study without invasive interventions, because it was usually conducted to improve the condition of the runners. The runners lived and trained near sea level (150 m) before the first training camp. Then, for the first training camp simulating the higher-altitude camp at low altitudes, they lived at 1800 m and trained at 1700 m twice a day, morning and afternoon, for 7 days. Afterwards, for the second training camp simulating the lower-altitude camp at low altitudes, they lived at 1550 m and trained at 1300 m twice a day, morning and afternoon, for 7 days. They then returned to near sea level. We sequentially collected saliva from these runners on the last 2 days during each camp which involved different exercise intensities in the morning and afternoon, modified as previously described^16^. Figure 1B details the relation between runner's altitudes and saliva collection times during 2 consecutive days during each training camp. On both days, saliva samples were collected at eight time points: upon waking (05:00), before morning exercise (06:00), after morning exercise (07:00), before breakfast (08:00), before lunch (12:00), before afternoon exercise (15:00), after afternoon exercise (16:00), and before dinner (18:00), as described previously^16^. On each training day at both camps, no differences were observed in the meteorological conditions; the temperature was around 20 °C and the relative humidity was 50–60% with fine weather. The runners were subjected to the following exercise program in the higher-altitude camp: 40-min fixed running in the morning and 50-min fixed running in the afternoon on day 1 (Higher-day 1); 8000-m fixed-distance running in the morning and uphill interval training with 8 sets of 200-m fast uphill running and light jogging in the evening on day 2 (Higher-day 2). The runners were subjected to the following exercise program in the lower-altitude camp: 50-min fixed running in the morning and 60-min fixed running in the afternoon on day 1 (Lower-day 1); 8000-m fixed-distance running in the morning and uphill interval training with 5 sets of 200-m fast uphill running and light jogging in the evening on day 2 (Lower-day 2). The runners drank enough water to prevent dehydration during these trainings.

### Physical examinations

The participants were weighed, and their body mass indexes were calculated as the weight divided by the squared height (kg/m^2^). After conducting interviews, no runners were found to use any medications or supplements. The runners used the Apple Watch Series 3 (Apple Japan Inc., Tokyo, Japan) during the camps. This allowed for the measurement of maximum pulse rate during each exercise and resting pulse rate at awakening and before dinner. The distance and duration of running during each exercise session was measured, and the running velocity was calculated as the distance divided by the duration (m/min)^15^. The Borg Rating of Perceived Exertion (RPE) scale^20^ was utilized to measure the runner's subjective exertion, breathlessness and fatigue after exercise. The runner's RPE was scored using a scale ranging from 6 to 20 and used in the analysis as a Borg scale score.

### Saliva collections and measurements of salivary cortisol concentrations

Sample collections and salivary cortisol concentration measurements were performed according to a previous study^16^. The runners were not allowed to brush their teeth, chew gum, or consume any food or drink except water, 15 min before the sample collection. Saliva samples were collected using Salivette^®^ cotton swabs (Sarstedt, Nümbrecht, Germany), centrifuged (1,500 × *g*) at 4 °C for 10 min, then immediately stored at − 80 °C until analysis. ECLIA measurements of salivary cortisol concentrations were performed using the Elecsys Cortisol II on the Cobas 8000 system (Roche Diagnostics K.K, Tokyo, Japan)^15,16^. The intra- and inter-assay coefficients of variation for salivary cortisol were 4.1% and 4.6%, respectively. The rate of change in the salivary cortisol concentration by exercise was calculated as the salivary cortisol concentration after exercise divided by the salivary cortisol concentration before exercise (%)^16^.

### Statistical analysis

The results of each measurement are expressed as the median values and corresponding 25th–75th percentile ranges. The Wilcoxon signed-rank test was utilized to identify statistically significant differences in variables between two different time points. A *p* value of < 0.05 was considered statistically significant. All statistical analyses were performed using SPSS Statistics, version 26.0 (IBM Corp., Armonk, NY, USA).

### Ethics approval and consent to participate

Written informed consent was obtained from all participants. This study was approved by the ethics committee of Gunma University Graduate School of Medicine (Approval number HS2018-140). All measurements were carried out by trained athletes and in accordance with the Declaration of Helsinki.

## Results

### Running intensity of each exercise program

Table 1 presents the characteristics of the participating female long-distance runners, whereas Table 2 presents the different running intensities of each exercise program during the two training camps. Because the exercise programs on days 1 and 2 were similar between the two camps, the exercise intensities of each program were compared. During the morning exercise on day 1, at the higher-altitude camp, the running velocity was significantly higher (*p* = 0.015) and the running distance and Borg scale scores were significantly lower (running distance, *p* = 0.029; Borg scale score, *p* = 0.047) than those at the lower-altitude camp. During the afternoon exercise on day 1, the running velocity was significantly higher at the higher-altitude camp (*p* = 0.003), but no differences were observed in other parameters between the two camps. During the morning exercise on day 2, the maximum pulse rate was significantly lower at the higher-altitude camp (*p* = 0.029), but no differences were observed in other parameters between the two camps. However, during the afternoon exercise on day 2, the running distance, Borg scale score, and maximum pulse rate were significantly higher at the higher-altitude camp (running distance, *p* = 0.002; Borg scale score, *p* = 0.005; maximum pulse rate, *p* = 0.036). When comparing the exercise intensities between the morning and afternoon on day 1, the running distance, running velocity, and Borg scale score were significantly higher during the afternoon exercise at the higher-altitude camp (running distance, *p* = 0.002; running velocity, *p* = 0.002; Borg scale score, *p* = 0.010), whereas the running distance was significantly higher during the afternoon exercise at the lower-altitude camp (*p* = 0.004). On day 2, the running distance and Borg scale score were significantly higher and the running velocity was significant lower during the afternoon exercise compared with those during the morning exercise at the higher-altitude camp (running distance, *p* = 0.002; Borg scale score, *p* = 0.004; running velocity, *p* = 0.002), whereas the running distance and velocity were significantly lower during the afternoon exercise at the lower-altitude camp (running distance, *p* = 0.002; running velocity, *p* = 0.003).

**Table 1:** Characteristics of female long-distance runners.

Characteristics	Value
Number	12
Age (year)	23.5 (19.5–26.0)
Height (cm)	160.0 (155.5–164.5)
Weight (kg)	45.5 (41.0–47.5)
Body mass index (kg/m^2^)	17.4 (17.0–17.9)

Data are expressed as median (25th–75th percentile).

**Table 2:** Running intensities of the exercise programs performed by female long-distance runners during the two camps.

	Altitude camp	Day 1		*p*_*day1*_	Day 2		*p*_*day2*_
		Morning exercise	Afternoon exercise		Morning exercise	Afternoon exercise	
Exercise program	Higher	40-min fixed running	50-min fixed running		8000-m fixed running	Uphill interval training	
	Lower	50-min fixed running	60-min fixed running		8000-m fixed running	Uphill interval training	
Running distance (m)	Higher	9700 (9250–10,150)*	12,790 (12,300–13,450)	0.002	8000 (8000–8000)	12,000 (9800–14,000)**	0.002
	Lower	11,585 (10,700–12,000)	13,145 (12,445–14,000)	0.004	8000 (8000–8000)	4700 (4200–5250)	0.002
Running velocity (m/min)	Higher	242.5 (224.8–247.7)*	252.0 (246.0–263.0)*	0.002	235.3 (228.6–238.9)	178.0 (153.9–215.4)	0.002
	Lower	224.2 (205.0–236.8)	219.1 (207.5–233.3)	0.477	235.3 (228.6–236.5)	168.0 (125.0–213.8)	0.003
Borg scale score	Higher	13.0 (12.0–15.0)*	15.0 (13.0–15.5)	0.010	13.0 (12.0–15.0)	16.0 (14.0–17.5)**	0.004
	Lower	14.0 (13.0–15.5)	13.5 (12.0–15.0)	0.119	13.0 (12.0–15.0)	13.0 (12.5–15.0)	0.524
Maximum pulse rate (beat/min)	Higher	175 (159–189)	182 (159–201)	0.182	170 (152–184)*	179 (165–192)*	0.167
	Lower	168 (153–198)	168 (158–181)	0.937	196 (164–210)	163 (154–187)	0.050

Data are expressed as median (25th–75th percentile).

*p < 0.05 and **p < 0.01 comparing variables between the higher- and lower-altitude camps using the Wilcoxon signed-rank test.

*p*_day1_ morning exercise vs. afternoon exercise on day 1 using the Wilcoxon signed-rank test.

*p*_day2_ morning exercise vs. afternoon exercise on day 2 using the Wilcoxon signed-rank test.

### Changes in the salivary cortisol concentrations in response to exercise within the circadian rhythms in each camp

**Figure 2:**
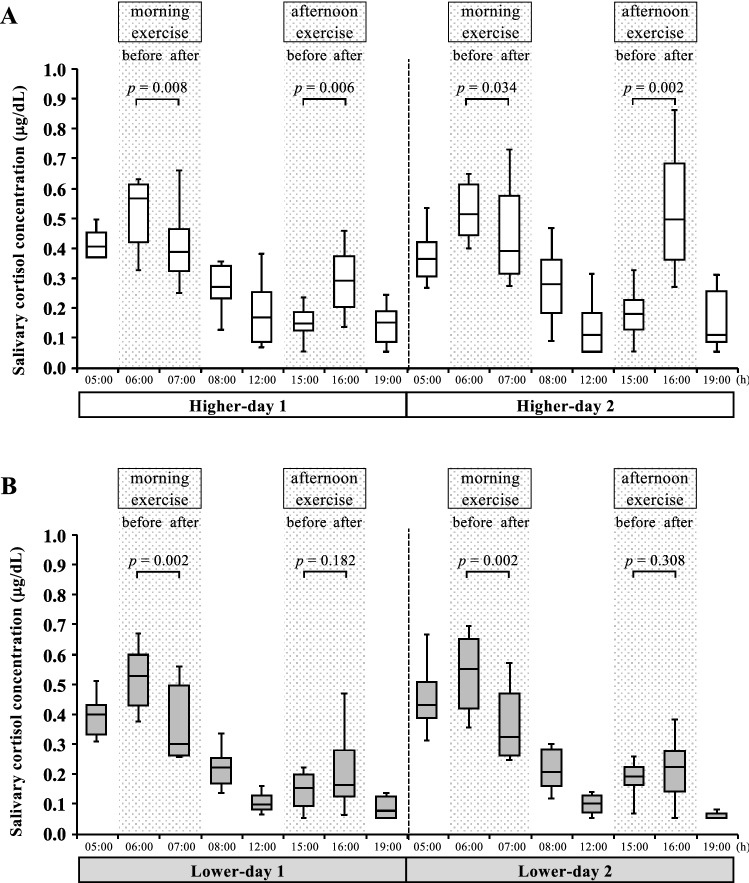
Changes in salivary cortisol concentrations in response to each exercise within the circadian rhythm on 2 consecutive days during the higher-altitude camp (**A**) and lower-altitude camp (**B**). The white box plots denote the cortisol concentration at the higher-altitude camp, whereas the gray box plots represent those at the lower-altitude camp. The gray dot squares denote the time of exercise at each camp. The significant differences between two time points of each exercise for runners were analyzed using the Wilcoxon signed-rank test. Higher-day 1, day 1 at the higher-altitude camp; Higher-day 2, day 2 at the higher-altitude camp; Lower-day 1, day 1 at the lower-altitude camp; Lower-day 2, day 2 at the lower-altitude camp.

Figure 2 presents the changes in the salivary cortisol concentrations in response to exercise during the last 2 days at both camps. The salivary cortisol concentrations peaked after waking and promptly decreased on both days at both camps. Within these circadian rhythms, the salivary cortisol concentrations significantly decreased after the morning exercise on both days at both camps but significantly increased after the afternoon exercise on 2 days at only the higher-altitude camp. These concentrations reached their lowest levels before dinner on both days at both camps. Table 3 presents that the differences in the resting pulse rates and salivary cortisol concentrations between the higher- and lower-altitude camps. The resting pulse rate before dinner was significantly higher on day 2 at the higher-altitude camp than on day 2 at the lower-altitude camp (*p* = 0.021). The salivary cortisol concentrations upon waking were significantly lower on both days at the higher-altitude camp than on day 2 at the lower-altitude camp (Higher-day 1 vs. Lower-day 2, *p* = 0.038; Higher-day 2 vs. Lower-day 2, *p* = 0.025). The concentrations before dinner were also significantly higher on both days at the higher-altitude camp than those on both days at the lower-altitude camp (Higher-day 1 vs. Lower-day 1, p = 0.026; Higher-day 1 vs. Lower-day 2, p = 0.005; Higher-day 2 vs. Lower-day 1, p = 0.012; Higher-day 2 vs. Lower-day 2, p = 0.003).

**Table 3:** Comparison of the variables between the higher- and lower-altitude camps in runners.

	Higher-altitude camp		Lower-altitude camp	
	Day 1	Day 2	Day 1	Day 2
Resting pulse rate at awakening (beat/min)	50 (46–55)	49 (44–52)	48 (44–52)	50 (42–57)
Resting pulse rate before dinner (beat/min)	65 (57–70)	65 (57–71)^†^	56 (52–68)	54 (48–63)
Salivary cortisol at awakening (μg/dL)	0.41 (0.37–0.45)^†^	0.36 (0.31–0.42)^†^	0.40 (0.33–0.43)	0.43 (0.39–0.50)
Salivary cortisol at peak(μg/dL)	0.57 (0.44–0.61)	0.51 (0.45–0.61)	0.49 (0.43–0.59)	0.55 (0.42–0.64)
Salivary cortisol before dinner (μg/dL)	0.15 (0.09–0.18)* ^††^	0.11 (0.09–0.25)* ^††^	0.08 (0.06–0.11)	0.05 (0.05–0.07)

Data are expressed as median (25th–75th percentile).

*p < 0.05 and **p < 0.01 comparing variables with day 1 at the lower-altitude camp using the Wilcoxon signed-rank test.

^†^*p* < 0.05 and ^††^*p* < 0.01 comparing variables with day 2 at the lower-altitude camp using the Wilcoxon signed-rank test.

### Rate of change in the salivary cortisol concentrations resulting from exercise in each camp

**Figure 3:**
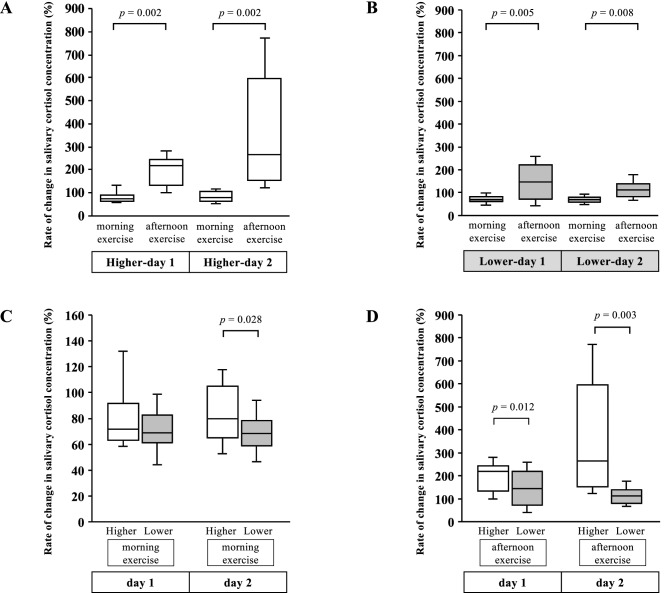
Comparison of the rate of change in the salivary cortisol concentration resulting from exercise between the morning and afternoon time points on days 1 and 2 at the higher-altitude camp (**A**), lower-altitude camp (**B**), between the morning time points on days 1 and 2 at the higher- and lower-altitude camps (**C**), and between the afternoon time points on days 1 and 2 at the higher- and lower-altitude camps (**D**). The white box plots denote the rates of change in the cortisol concentration at the higher-altitude camp, whereas the gray box plots represent those at the lower-altitude camp. The significant differences between two time points of exercise for runners were analyzed using the Wilcoxon signed-rank test. Higher-day 1, day 1 at the higher-altitude camp; Higher-day 2, day 2 at the higher -altitude camp; Lower-day 1, day 1 at the lower-altitude camp; Lower-day 2, day 2 at the lower-altitude camp.

Figure 3 presents the comparison of the rate of change in the salivary cortisol concentrations after each exercise at both camps. The rates of change in salivary cortisol concentrations were significantly lower during the morning exercise than during the afternoon exercise on both days at each camp (Higher-day 1, *p* = 0.002; Higher-day 2, *p* = 0.002; Lower-day 1, *p* = 0.005; Lower-day 2, *p* = 0.008; Fig. 3A,B). After the morning exercise, the rate of change in the salivary cortisol concentrations was significantly higher on day 2 at the higher-altitude camp than on day 2 at the lower-altitude camp (*p* = 0.028; Fig. 3C). After the afternoon exercise, the rate of change in the salivary cortisol concentrations was significantly higher on both days at the higher-altitude camp than on both days at the lower-altitude camp (Higher-day 1 vs. Lower-day 1, *p* = 0.012; Higher-day 2 vs. Lower-day 2, *p* = 0.003; Fig. 3D).

## Discussion

In this study, we demonstrated whether the stress responses of runners in training camps at different altitudes could be evaluated via sequential saliva collection and automated salivary cortisol measurement. These methods were able to detect the basal levels and exercise-induced changes in the salivary cortisol within the runners' circadian rhythms at each altitude camp. The basal salivary cortisol concentrations before dinner were significantly higher at the higher-altitude camp than at lower-altitude camp. The rate of change in the salivary cortisol concentrations during the afternoon exercise on days 1 and 2 and the indicators of exercise intensity were significantly higher at the higher-altitude camp than at the lower-altitude camp. Moreover, the rate of change in the salivary cortisol concentrations during the morning exercise on day 2 was significantly higher at the higher-altitude camp than at lower-altitude camp; no differences were observed in the exercise programs and intensities, such as the running distances, velocities, and Borg scale scores.

There have been contradictory reports with regard to the effects of altitude training on cortisol secretions. In male elite climbers, the resting serum cortisol and plasma adrenocorticotropic hormone (ACTH) levels taken at 07:00–07:30 did not change at 5200 m extreme altitude camp compared with those at sea level^6^. In male and female elite skiers, no significant differences were observed in resting salivary cortisol concentrations taken at 07:00–08:00 between a control group training and living at an altitude of 1200 m and another group training at 1200 m but living at a simulated altitudes of 2500 m, 3000 m, and 3500 m for 6 days in hypoxic rooms^7^. In contrast, the basal concentrations of serum cortisol in the morning after 3–4 days at 4350 m were elevated compared with those at sea level in healthy men, but this was not statistically significant^8^. In the present study, a significant difference was observed in the basal salivary cortisol concentrations before dinner between the two camps with an altitude difference of approximately 300 m, even at the relatively low altitudes of 1300–1800 m. These differences may be due to the fact that the lowest cortisol levels were compared in the evening, whereas the previous studies compared the concentrations in the morning^6–8^. Another reason may be that the athletes were acclimatized to these altitudes when transitioning from the first higher-altitude camp to the second lower-altitude camp. Overtraining syndrome causes a reduced cortisol response to exercise and changes in the circadian rhythms of cortisol, including low resting levels and peak loss after waking^21,22^. In the present study, the runners' salivary cortisol concentrations were lower in the evening, but peaked after waking and increased after high-intensity exercise. The low levels of salivary cortisol in the evenings indicated that the runners were not suffering from overtraining syndrome but rather were able to adapt to the higher altitudes. Although further analysis under higher-altitude conditions is required, it may be more useful to evaluate the cortisol levels in the evening rather than in the morning using serum or saliva for assessing the acclimatization to the several altitude environments among athletes.

Regarding the acute response to exercise, the increase in cortisol levels after interval training at 09:00 exhibited an insignificant trend toward higher values at an altitude of 1800 m when compared with that observed at near sea levels in highly trained endurance athletes^9^. Another study found that the serum cortisol levels significantly increased after resistance training at 70% of the maximum strength under 13% hypoxic conditions from 08:00 to 11:30 but not under normoxic conditions in healthy male subjects^10^. Conversely, the serum cortisol levels did not change after resistance training at 50% of the maximum strength under hypoxic and normoxic conditions^11^. In the present study, the rates of change in the salivary cortisol concentration after the morning exercise were significantly lower than those after the afternoon exercise on both days at each altitude camp due to the influence of the circadian rhythm, which was validated by previous reports^12,16^. In contrast , the comparison of the rates of change in cortisol concentration during exercise at the same time on different days, whether in the morning or in the afternoon, was effective in evaluating the stress responses, as previously described^16^. In the present study, we were able to detect the difference in the rates of change in the salivary cortisol concentration during exercise with the same program at different altitudes, even in the morning. Moreover, we could detect the increase in stress due to the differences in altitude and exercise intensity, more clearly in the afternoon, by assessing the rate of change in salivary cortisol concentrations. It was revealed that an increase in the training altitude of approximately 400 m at the low altitude of 1300–1700 m with high-intensity exercise resulted in an increase in cortisol secretion in both morning and afternoon. This result validates the previous studies, where the serum cortisol concentrations were acutely elevated by high-intensity exercise with higher VO_2 max_^4,5^. However, we did not measure the oxygen saturation (SpO_2_) or VO_2 max_ as the oxygen tolerability of runners for different exercise intensities. Future studies should evaluate the relationships between the changes in salivary cortisol concentrations and these oxygen tolerability markers in response to endurance exercises at different altitudes. Additionally, the range of the runners' salivary cortisol concentrations was broad, especially after afternoon exercise on day 2 at the higher-altitude camp, shown in Fig. 2. This broad range suggested individual differences in stress responses induced by the altitude training in the runners. Therefore, the cortisol concentration measurement technique described in this study which uses continuous saliva collection during the circadian rhythms would be more useful as a personalized conditioning tool to detect the differences in stress responses under various environments for an individual athlete, rather than as a statistical analysis tool for a large number of athletes.

This study has several limitations. First, the sample size was relatively small. We focused on enrolling well-trained female runners with standardized meal and sleep times during the two consecutive camps. Second, the sequential saliva collections and measurements of salivary cortisol concentration were not performed at sea level. The temperatures were around 20 °C during both altitude camps, but the temperature during the same period was as high as 30–40 °C at near sea level where the runners lived and trained. Previous research found that the salivary cortisol levels detected in a maximal progressive test using a cycle ergometer were significantly higher under the hot conditions (40 °C) than under normal conditions (22 °C) in nine young healthy men^23^. Therefore, it was impossible in this study to collect saliva at near sea level without the stress resulting from high temperatures. Future study should utilize hypoxic rooms, in which the environmental conditions, including temperature, are standardized. Additionally, more runners should be enrolled, specifically males.

## Conclusions

Measurement of the salivary cortisol levels within the circadian rhythm led to the detection of the changes in the stress response due to the same intensity exercise at different altitudes, even in the morning. Additionally, evening resting salivary cortisol levels can be used to assess athletes' acclimatization to high altitudes. The combination of sequential saliva collection and automated cortisol measurements may be useful for assessing adaptation disorders and excessive exercise stress, and also may help develop adequate altitude training programs for athletes.

## Data Availability

The datasets used and/or analyzed during the current study are available from the corresponding author on reasonable request.

## Abbreviations

ACTH: Adrenocorticotropic hormone
ECLIA: Electrochemiluminescence immunoassay
ELISA: Enzyme-linked immunosorbent assay
RPE: Rating of perceived exertion
SpO_2_: Oxygen saturation
VO_2 max_: Maximal oxygen consumption
